# Bidirectional Associations between Popularity, Popularity Goal, and Aggression, Alcohol Use and Prosocial Behaviors in Adolescence: A 3-Year Prospective Longitudinal Study

**DOI:** 10.1007/s10964-020-01308-9

**Published:** 2020-08-31

**Authors:** Sarah T. Malamut, Yvonne H. M. van den Berg, Tessa A. M. Lansu, Antonius H. N. Cillessen

**Affiliations:** 1 INVEST Research Flagship, Department of Psychology, University of Turku, Turku, Finland; 2 Behavioural Science Institute, Radboud University, Nijmegen, The Netherlands

**Keywords:** Popularity, Popularity goal, Aggression, Alcohol use, Prosocial behavior, Adolescence

## Abstract

Adolescents’ popularity and popularity goal have been shown to be related to their aggression and alcohol use. As intervention efforts increasingly aim to focus on prosocial alternatives for youth to gain status, it is essential to have a comprehensive understanding of how popularity and popularity goal are associated with aggression and substance use as well as prosocial behaviors over time. The current study examined the bidirectional associations of aggression (overt and relational aggression), alcohol use, and prosocial behavior with popularity and popularity goal in adolescence across 3 years using cross-lagged panel analyses. Participants were 839 Dutch adolescents (*M*_*age*_ = 13.36, *SD* = 0.98; 51.3% girls). The results indicated that popularity was consistently positively associated with popularity goal, but popularity goal did not significantly predict subsequent popularity. Popularity positively predicted elevated aggression and alcohol use, but lower levels of prosocial behavior. For the full sample, alcohol use and overt aggression in grade 7 both predicted subsequent popularity in grade 8. However, when considering gender differences, overt aggression no longer was a significant predictor of popularity. These results were discussed in terms of the dynamic interplay between popularity, popularity goal, and behaviors, and in terms of implications for prevention and intervention efforts.

## Introduction

A key developmental change in early adolescence is the growing attention for peer relationships (e.g., Brown and Larson 2009). When youth become more interested in their interactions with peers, they also begin to strive more intensely for popularity in the peer group, with popularity goal typically peaking in early adolescence (e.g., Dawes and Xie 2017; LaFontana and Cillessen 2010). There is growing evidence that youth’s actual level of popularity and their motivations to be (more) popular are independently predictive of youth’s behaviors. Being popular as well as popularity goal are both concurrently associated with risky behaviors, such as aggression and alcohol use, but also with prosocial behaviors (e.g., Cillessen et al. 2014; Dumas et al. 2017). Whereas several studies have demonstrated the interplay between popularity goals and actual popularity status in predicting behavior concurrently (e.g., Cillessen et al. 2014; van den Broek et al. 2015), there is a lack of longitudinal studies with more than two time points. Furthermore, the few longitudinal studies on popularity, goals, and behavior have exclusively focused on aggression and/or alcohol use, and have not included prosocial behaviors (e.g., Dawes and Xie 2014). The goal of the current study is therefore to fill this gap by examining the bidirectional associations among adolescents’ popularity, popularity goal, and their aggression (overt aggression, relational aggression), alcohol use, and prosocial behavior in a 3-year prospective study.

Insight into the longitudinal bidirectional links of status and status goals with behaviors is important for several reasons. First, popularity and popularity goal may both predict and be reinforced by adolescents’ behaviors (Dumas et al. 2017). For example, youth who are popular or wish to be popular may be more likely to engage in aggression and health-risk behaviors (e.g., drinking alcohol). These behaviors may in turn result in greater popularity or popularity goal, due to peer reinforcement or the acquisition of social resources. This potential cyclical relation may lead to negative outcomes in terms of elevated aggression and extreme or age inappropriate use of alcohol, and these behaviors are known to affect concurrent and long-term developmental outcomes. Second, although research often has focused on the associations between status or status goals and aggression or health-risk behaviors, it is also important to consider prosocial behaviors. Previous research has indicated that individuals may use aggressive or collaborative strategies to gain or maintain status (e.g., Cheng et al. 2013; Hawley 2003). Third and relatedly, some researchers have posited that interventions should focus on establishing prosocial ways for youth to reap the benefits associated with status (e.g., notoriety; Ellis et al. 2016). To do so effectively, it is essential to understand longitudinal associations between prosocial behaviors, popularity, and popularity goal. Fourth and finally, the behaviors of popular youth also influence the wider peer group. Prior studies have shown that popular adolescents have more influence on risky and aggressive behaviors than other classmates (e.g., Cohen and Prinstein 2006; Teunissen et al. 2012). In order to prevent or intervene in these broader social network processes, it is important to understand the role of youth’s popularity and popularity goal.

### Popularity and Popularity Goal

A defining characteristic of the social hierarchy in the peer group is that not everyone can achieve high status (e.g., Hawley 1999). However, not everyone strives for this limited resource to the same extent, as youth differ in how much they value high status, such as being popular. It seems that adolescents who are more popular than their peers are the ones who tend to be more motivated to remain or become even more popular, as previous studies have found a moderate association between actual popularity and popularity goal (e.g., Dawes and Xie 2017). As such, high-status youth who have experienced the benefits of popularity (e.g., resources, attention: Hawley 1999, 2003) may subsequently have a higher popularity goal. However, having a strong popularity goal by itself does not necessarily lead to actual heightened status later in time, as not all youth who aspire to be popular have the resources or skills to actually become popular (Dawes and Xie 2014). Moreover, as youth with the highest popularity goal may already have high status, there may be a ceiling effect. Therefore, popularity was expected to predict elevated popularity goal, rather than popularity goal predicting elevated popularity.

### Popularity, Popularity Goal, and Aggression, and Alcohol Use

High levels of popularity and popularity goal have been linked concurrently and uniquely with aggression (Cillessen et al. 2014). Across time, popularity has been shown to typically be a predictor, rather than an outcome, of aggression (Ojanen and Findley-Van Nostrand 2014; Prinstein and Cillessen 2003). However, some research suggests that relational aggression (e.g., damaging a peer’s reputation) in particular can predict increases in popularity (e.g., Cillessen and Mayeux 2004; Rose et al. 2004). Taken together, previous research suggests that aggression is used by adolescents who already are popular to *maintain* their status, and may or may not be used to *become* popular (e.g., Hawley 2003; Prinstein and Cillessen 2003). Youth who already are popular may engage in overt forms of aggression (e.g., hitting, kicking peers) to maintain their status or demonstrate their dominance to the peer group (e.g., Cillessen and Mayeux 2004). Furthermore, adolescents may effectively use other more subtle forms of aggression, such as rumor spreading, to challenge the reputations of social competitors and gain popularity status through it (e.g., Rose et al. 2004). Therefore, popularity was expected to be positively associated with future overt and relational aggression. Given that some studies found that relational aggression in particular predicted popularity (e.g., Cillessen and Mayeux 2004), it was also hypothesized that relational aggression would predict elevated popularity.

In addition to aggression, high-status youth are likely to engage in health-risk behaviors (Schwartz and Gorman 2011). For example, Mayeux et al. (2008) found that popularity in 10th grade predicted increases in alcohol use in 12th grade. This is concerning, as popular youth are visible and influential in the peer group; the behaviors of high-status adolescents are salient to their peers and influence the perceived norm for acceptable behavior in the peer group (e.g., Dijkstra et al. 2008). For adolescents, alcohol use is closely intertwined with social activities and the desire to fit in (e.g., Teunissen et al. 2012). Dumas et al. (2017) found that drinking predicted increases in self-reported popularity. Despite these findings, it is unclear whether drinking predicts increases in being seen as popular by peers. Being willing to drink and eschewing adult norms may be a way for youth to show autonomy or maturity (e.g., “the maturity gap”; Gommans et al. 2016; Moffitt 1993). In this way, alcohol use may predate popularity. In addition, popular youth may have more opportunities (e.g., parties) to be exposed to alcohol than unpopular youth (e.g., Schwartz and Gorman 2011). Therefore, bidirectional, positive associations between alcohol use and popularity were expected.

Distinct from youth’s actual popularity, their popularity goals may also impact their likelihood to engage in aggression or risk behaviors. As aggression and drinking alcohol likely is associated with popularity in the minds of adolescents, those who want to be popular may use aggression or alcohol to advance their status (e.g., Dawes and Xie 2014; Ellis and Wolfe 2009). This line of reasoning is consistent with popularity goals being associated with these behaviors concurrently (e.g., Cillessen et al. 2014), as well as longitudinally (e.g., Dumas et al. 2017). Therefore, it was hypothesized that popularity goal would be positively associated with overt aggression, relational aggression, and alcohol use over time.

### Popularity, Popularity Goal, and Prosocial Behavior

Although there are well-documented links of popularity with aggression and risk behaviors, it is also important to consider prosocial behaviors. Consistent with the dominance-prestige model (e.g., Cheng et al. 2013), individuals may use either aggressive or collaborative strategies to ascend the social hierarchy. Moreover, past research has identified a subset of popular youth who are prosocial, rather than aggressive (i.e. “models”; de Bruyn and Cillessen 2006; Rodkin et al. 2000). Indeed, numerous studies find modest concurrent associations between popularity and prosocial behavior in adolescence (e.g. Li and Wright 2014), although other studies have not found such an association (e.g., Cillessen et al. 2014). However, information regarding the longitudinal associations between popularity and prosocial behavior is more scarce. One study with Chinese adolescents demonstrated bidirectional associations between popularity and elevated levels of prosocial behavior over time (Lu et al. 2018). In an American sample, popularity was found to predict increased online prosocial behavior (Wright 2014). Given these findings and the potential of prosocial behavior to support youth’s position as a “model” popular peer, it was hypothesized that there would be concurrent and longitudinal associations between popularity and prosocial behavior.

Whereas there is empirical support for a link between popularity and prosocial behavior, less is known regarding the association between popularity goal and prosocial behavior. Some research suggests that popularity motivations are negatively associated with concurrent prosocial behaviors (van den Broek et al. 2015), whereas another study found no association between popularity goal and prosocial behavior (Li and Wright 2014). In general, the findings seem to indicate that popularity may be associated with a range of both prosocial and aggressive behaviors, but popularity goal is more strongly associated with aggression and alcohol use than prosocial behavior (van den Broek et al. 2015). As youth with a strong popularity goal do not typically seem to choose prosocial behavior as a way to achieve that goal, we did not expect significant concurrent or prospective links between popularity goal and prosocial behavior. In other words, youth who strive for popularity do not typically seem to consider prosocial behaviors as an effective means to become (more) popular.

### Gender Differences

It is important to consider potential gender differences in the associations between popularity, popularity goal, and behaviors. Thus far, there is not much evidence of mean level gender differences in popularity (e.g., Cillessen et al. 2014; Malamut et al. 2020). As much of the literature on popularity goals has not directly tested for mean level gender differences (e.g., Dawes and Xie 2014, 2017; Li and Hu 2018), it is less clear whether boys and girls differ with regard to popularity goals. However, there is some evidence that gender differences in overt aggression (e.g., Card et al. 2008), relational aggression (e.g., Prinstein and Cillessen 2003), alcohol use (e.g., La Greca et al. 2001), and prosocial behavior (e.g., Cillessen et al. 2014) exist. In general, boys appear to be more likely to use overt or direct forms of aggression than girls (Card et al. 2008). Whereas some research has found that girls are more likely to be relationally aggressive than boys, other studies have found negligible differences (e.g., Card et al. 2008). Boys typically consume more alcohol than girls (Engels et al. 2006; La Greca et al. 2001), whereas girls seem to be viewed by peers as more prosocial than boys (Cillessen et al. 2014; Van der Graaff et al. 2018). However, there are few indications that the longitudinal associations between popularity, popularity goal, and behaviors are moderated by gender (e.g., Dumas et al. 2017). Nonetheless, given that boys and girls may engage in these behaviors at different rates, potential gender differences in the longitudinal associations between popularity, popularity goal, and behaviors were explored.

## Current Study

The current study builds on past research examining the associations between popularity, popularity goal, and behavior (overt aggression, relational aggression, alcohol use, prosocial behavior) in a 3-year prospective longitudinal design. The longitudinal data allowed us to examine the temporal direction of associations, including bidirectional effects, which can inform future prevention efforts. Popularity was expected to be a stronger predictor of popularity goal, rather than popularity goal predicting subsequent popularity. Strong associations of both popularity and popularity goal with subsequent overt and relational aggression were hypothesized. There is mixed evidence for aggression predicting subsequent popularity or popularity goal, which we aimed to further clarify. Popularity goal was expected to predict alcohol use, and popularity was expected to be both a predictor and outcome of alcohol use. The current study also investigated the longitudinal links of prosociality with popularity and popularity goal, both of which are less established. Potential gender differences were considered when the aforementioned associations were examined.

## Method

### Participants

Participants were recruited as part of the Kandinsky Longitudinal Study (KLS), a longitudinal study that started in 2010 to identify youth at risk for socio-emotional adjustment difficulties (van den Berg et al. 2019). For the current study, data from participants in grades 7 to 9 during waves 5 through 7 (i.e., years 2014–2016) was used. At T1 (2014), there were 839 participants (51.3% girls, *M*_*age*_ = 13.36, *SD* = 0.98; *n*_Grade7_ = 286, *n*_Grade8_ = 260, *n*_Grade9_ = 293). At T2 (2015), there were 833 participants (50.1% girls, *M*_*age*_ = 13.66, *SD* = 1.02; *n*_Grade7_ = 273, *n*_Grade8_ = 283, *n*_Grade9_ = 277). At T3 (2016), there were 812 participants (49.9% girls, *M*_*age*_ = 13.63, *SD* = 0.93; *n*_Grade7_ = 265, *n*_Grade8_ = 267, *n*_Grade9_ = 280). At each wave, the majority of participants were born in the Netherlands (ranging from 95.2 to 96%) and had parents who were born in The Netherlands (ranging from 81.9 to 85.8%). For the analyses, participants across all three waves were grouped by grade (i.e., grade 7 to 9).

### Procedure

The head of the school explicitly requested the research to be conducted each year, and as such took responsibility for parental consent procedures. The school gave parents a detailed letter outlining the goal and procedures of the data collection. Parents were informed they could exclude their child from participation. No parents objected to the participation of their son or daughter. Adolescents were also informed and asked for active assent at the beginning of the assessment. None of the students declined to participate at any stage of the assessment. This procedure was approved by the Institutional Review Board of the Behavourial Science Institute at Radboud University (Protocol Number: ECG2012-2505-038; Project Title: “Sociometry as a method to measure social relationships among children and adolescents”).

Each year, a combination of peer nominations and self-reports were assessed via a computerized questionnaire (see van den Berg and Cillessen 2013, for the detailed procedure) during 45 to 60 min classroom sessions. Each year, researchers explained the goal and procedure of the study prior to administration of the questionnaires. Students were reassured that the data would be processed anonymously and confidentially. Participants were instructed to not share answers with classmates and to respond honestly to all questions. They were prohibited from speaking to classmates during the assessment, but were allowed to ask the researchers questions or stop participating at any time.

### Measures

To measure popularity, aggression, and prosocial behavior, peer nominations were used. For each nomination question, participants were able to nominate an unlimited number of same- and cross-gender classmates. Participants were required to make a minimum of one nomination, and could not nominate themselves for any item as their name was not presented on the screen.

#### Popularity

Participants nominated their classmates who were “most popular” and “least popular.” The total number of nominations that adolescents received for each item was counted and standardized within classrooms (Cillessen and Marks 2011). Popularity was then calculated by subtracting the “least popular” scores from the “most popular” scores, again standardizing the resulting difference score within classrooms.

#### Overt and relational aggression

To measure overt aggression, participants were asked “who from your class push, kick, or hit others?”. Relational aggression was measured by asking “who from your class say mean things or gossip about others?”. Nominations received for each item were counted and standardized within classrooms.

#### Prosocial behavior

Participants were asked “who from your class is often willing to help others?”. The number of nominations received for this item was counted and standardized within classrooms.

#### Alcohol use (self-report)

To assess alcohol use, participants were asked “in the last 30 days, on how many days did you drink alcohol?” (e.g., Gommans et al. 2016). Adolescents responded using a Likert scale ranging from 0 (*never*) to 6 (*all 30 days*).

#### Popularity goal (self-report)

Adolescents were asked *“*how important is it for you to be popular in your class?” (e.g., Dawes and Xie 2014). Responses could range from −3 (*not important at all*) to +3 (*very important*).

## Results

### Descriptive Statistics

Table 1 depicts the means and standard deviations for all variables for the full sample, and separately for boys and girls. In all grades, boys were perceived as more overtly aggressive than girls, whereas girls were nominated more often as prosocial. In grade 7 only, boys reported higher levels of alcohol use than girls. In grades 8 and 9, girls scored higher on relational aggression than boys. The only gender difference in popularity and popularity goal regarded popularity in grade 9, with boys scoring higher on popularity than girls.

**Table 1:** Means and standard deviations separate for boys and girls

	Full sample	Boys	Girls	
Variable	*M (SD)*	*M (SD)*	*M (SD)*	*t*
Grade 7				
Popularity	0.00 (1.66)	0.07 (1.76)	−0.07 (1.55)	1.14
Popularity goal	0.01 (1.49)	0.09 (1.45)	−0.06 (1.52)	1.41
Overt aggression	−0.00 (0.98)	0.38 (1.19)	−0.38 (0.47)	12.01***
Relational aggression	0.00 (0.98)	−0.06 (0.92)	0.06 (1.04)	−1.78
Alcohol use	0.05 (0.24)	0.07 (0.30)	0.02 (0.17)	2.79**
Prosocial behavior	0.00 (0.98)	−0.31 (0.89)	0.31 (0.97)	−9.47***
Grade 8				
Popularity	0.00 (1.64)	0.10 (1.70)	−0.10 (1.57)	1.74
Popularity goal	−0.08 (1.45)	−0.08 (1.44)	−0.09 (1.45)	0.09
Overt aggression	−0.00 (0.98)	0.38 (1.18)	−0.38 (0.50)	11.76***
Relational aggression	−0.00 (0.98)	−0.25 (0.75)	0.24 (1.11)	−7.42***
Alcohol use	0.11 (0.44)	0.13 (0.48)	0.09 (0.40)	1.34
Prosocial behavior	0.01 (0.98)	−0.30 (0.86)	0.30 (0.10)	−9.20***
Grade 9				
Popularity	−0.01 (1.65)	0.19 (1.63)	−0.21 (1.64)	3.53***
Popularity goal	0.09 (1.43)	0.13 (1.43)	0.05 (1.43)	0.73
Overt aggression	−0.01 (0.96)	0.43 (1.15)	−0.43 (0.44)	13.92***
Relational aggression	−0.01 (0.97)	−0.17 (0.91)	0.14 (1.00)	−4.74***
Alcohol use	0.31 (0.68)	0.35 (0.78)	0.28 (0.57)	1.47
Prosocial behavior	0.02 (0.98)	−0.30 (0.87)	0.32 (0.98)	−9.56***

**p < 0.01; ***p < 0.001

Bivariate correlations between all variables are shown in Table 2 by gender. Due to the number of tests, correlations were only considered significant at *p* < 0.001. Two-sided Fisher’s Z-tests were used to examine whether the correlations were significantly different between boys and girls. The stabilities of most variables were significant for both genders, with a few exceptions. Alcohol use in grade 7 was not significantly associated with grade 8 alcohol use for girls or grade 9 alcohol use for girls and boys. Furthermore, for girls only, grade 7 and 8 overt aggression were not significantly associated with grade 9 overt aggression. Thus, with the exception of alcohol use, most constructs were highly stable across all three grades.

**Table 2:** Bivariate associations between study variables separate for boys and girls

	Grade 7						Grade 8						Grade 9					
Variable	1	2	3	4	5	6	7	8	9	10	11	12	13	14	15	16	17	18
Grade 7																		
1. Popularity	–	0.22***	0.26***	0.43***	0.05	0.21***	0.80***	0.16	0.20***	0.51***	0.09	0.06	0.68***	0.24	0.01	0.39***	0.27	0.34***
2. Popularity goal	0.24***	–	0.11	0.12	0.06	−0.01	0.23***	0.51***	0.17	0.18	0.09	−0.04	0.23	0.44***	0.00	0.10	0.07	0.19
3. Overt aggression	0.20***	0.10	–	0.40***	−0.03	−0.17***	0.28***	0.06	0.50***	0.29***	0.04	−0.14	0.17	0.04	**0.03**	0.42***	0.11	−0.26
4. Relational aggression	0.40***	0.10	0.47***	–	0.08	−0.24***	0.42***	−0.00	0.24***	0.49***	0.10	−0.22***	0.35***	0.04	0.14	0.51***	0.31***	−0.05
5. Alcohol use	0.14	0.01	0.22***	0.25***	–	−0.09	0.07	0.11	−0.00	0.08	**0.08**	−0.10	0.05	0.06	a	0.12	**0.18**	−0.02
6. Prosocial behavior	0.17***	0.02	−0.33***	−0.22***	−0.14	–	0.14	0.00	−0.13	−0.15	−0.04	0.55***	0.18	0.01	−0.12	−0.17	−0.16	0.53***
Grade 8																		
7. Popularity	0.80***	0.20	0.17	0.31***	0.17	0.12	–	0.26***	0.24***	0.61***	0.10	0.09	0.76***	0.29***	0.07	0.54***	0.36***	0.18
8. Popularity goal	0.29***	0.49***	−0.03	0.02	0.02	0.02	0.31***	–	0.02	0.16	0.05	−0.06	0.27***	0.52***	−00.11	0.08	0.25***	0.03
9. Overt aggression	0.16	0.07	0.54***	0.32***	0.17	−0.25***	0.29***	−0.00	–	0.35***	0.00	−0.14	0.13	−0.08	**0.06**	0.29***	−0.04	−0.14
10. Relational aggression	0.27***	0.08	0.18	0.38***	0.17	−0.20***	0.40***	0.10	0.50***	–	0.09	−0.26***	0.52***	0.18	0.09	0.60***	0.37***	−0.13
11. Alcohol use	0.15	0.06	0.13	0.04	0.28***	0.00	0.22***	0.10	0.12	0.20***	–	−0.06	0.07	−0.04	−0.01	0.11	0.31***	−0.03
12. Prosocial behavior	−0.04	−0.02	−0.21***	−0.25***	−0.14	0.56***	−0.05	0.00	−0.35***	−0.32***	−0.10	–	0.14	−0.07	−0.11	−0.15	−0.10	0.63***
Grade 9																		
13. Popularity	0.68***	0.20	0.04	0.29	0.09	0.29***	0.76***	0.21	0.25***	0.31***	0.16	−0.06	–	0.36***	0.17***	0.58***	0.35***	0.16***
14. Popularity goal	0.21	0.53***	−0.04	0.04	0.02	−0.00	0.13	0.54***	0.03	0.09	0.00	−0.00	0.26***	–	0.04	0.21***	0.19***	−0.09
15. Overt aggression	0.18	0.20	0.45***	0.23	0.07	−0.15	0.36***	0.17	0.48***	0.39***	0.21***	−0.21***	0.42***	0.10	–	0.33***	0.03	−0.08
16. Relational aggression	0.30***	0.10	0.11	0.40***	0.04	−0.02	0.43***	0.12	0.30***	0.49***	0.12	−0.16	0.49***	0.12	0.56***	–	0.30***	−0.18***
17. Alcohol use	0.15	0.04	0.04	0.01	**0.10**	0.01	0.26***	0.12	0.17	0.09	0.28***	−0.10	0.30***	0.11	0.17***	0.09	–	−0.10
18. Prosocial behavior	−0.01	−0.14	−0.18	−0.23	−0.11	0.39***	−0.05	−0.15	−0.23***	−0.21***	−0.07	0.46***	0.00	−0.00	−0.27***	−0.23***	−0.14	–

Due to the large number of correlations, only effects significant at ***p < 0.001 are presented. Significant correlations were tested with a two-sided Fisher’s Z-test; correlations underlined are significantly different between girls (above the diagonal) and boys (below the diagonal). Coefficients in bold represent stabilities

^a^This correlation could not be calculated because all girls with overt aggression scores in G9 scored a “0” on alcohol use in G7

In each grade, there was a modest positive association between popularity and popularity goal for both genders (*rs* ranging from 0.22 to 0.36). In grade 7, popularity was positively associated with all grade 7 behaviors, except for alcohol use, for both genders. In grade 8, popularity was positively associated with overt and relational aggression for both genders, and also with alcohol use for boys. In grade 9, popularity was positively correlated with all behaviors for girls, but only with aggression and alcohol use for boys. Popularity goal in grades 7 and 8 were not significantly correlated with any grade 7 or 8 behaviors, respectively, for either gender. In grade 9, popularity goal was positively associated with relational aggression and alcohol use for girls only. Thus, in all grades, popularity was more consistently associated with aggression, alcohol use and prosocial behaviors than popularity goal.

### Cross-Lagged Panel Analyses

**Fig. 1:**
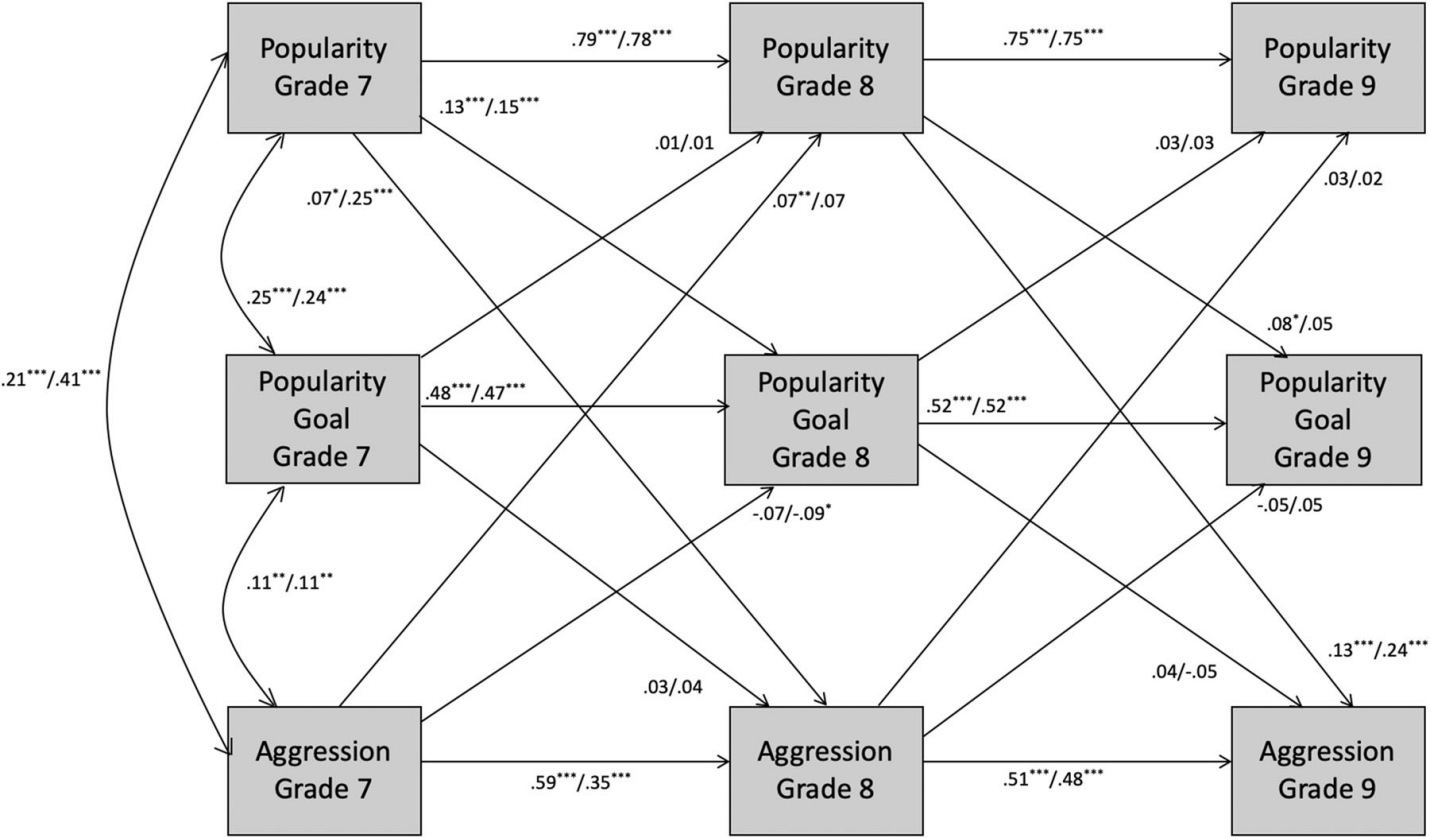
Longitudinal associations among popularity, popularity goal, and aggression for the full sample. For each path, the standardized estimates are shown for the models including overt aggression and relational aggression, respectively. Correlated errors are not shown for clarity of presentation. *p < 0.05; **p < 0.01; ***p < 0.001

**Fig. 2:**
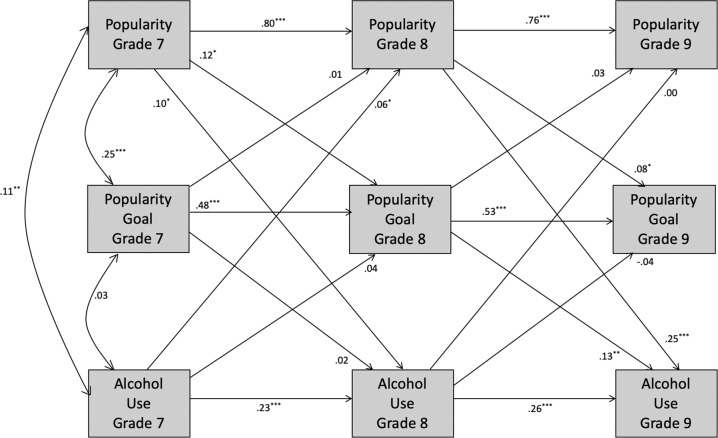
Longitudinal associations among popularity, popularity goal, and alcohol use for the full sample. Correlated errors are not shown for clarity of presentation. *p < 0.05; **p < 0.01; ***p < 0.001

**Fig. 3:**
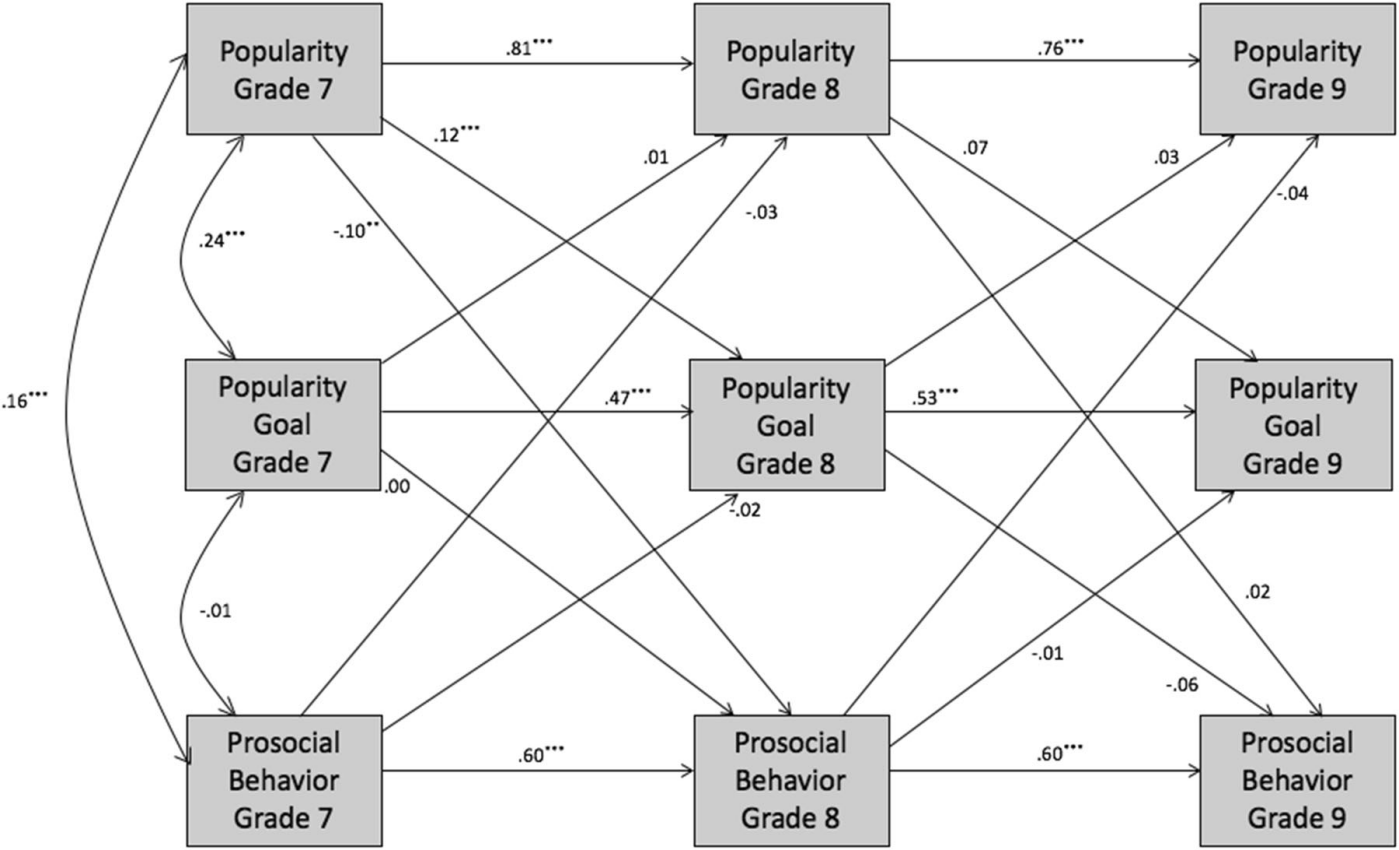
Longitudinal associations among popularity, popularity goal, and prosocial behavior for the full sample. Correlated errors are not shown for clarity of presentation. *p < 0.05; **p < 0.01; ***p < 0.001

Cross-lagged panel analyses were conducted to examine the longitudinal associations between popularity, popularity goal, and behavior from grade 7 to 9. A separate model was run for each behavior (overt aggression, relational aggression, alcohol use, prosocial behavior; see Figs. 1–3) in Amos 24.0. In each model, stability paths were specified from grades 7 to 8 and 8 to 9 for each construct. Predictive paths from each construct in grade 7 to the other constructs in grade 8 were specified, and similarly from grade 8 to grade 9. Full-information maximum likelihood (FIML) was specified to include all available observations, avoiding biases from only including participants with complete data. Little’s (1988) MCAR test indicated that the missing values were missing completely at random, χ^2^ = 230.273, df = 252, p = 0.83. To explore gender differences, each model was run as a two-group model for boys and girls separately and fit was compared with specific paths constrained and unconstrained (e.g., Cillessen and Mayeux 2004; see Appendix for additional information on model fit indices).

#### Overt aggression

The fit of the model (Fig. 1) was good, χ^2^(9) = 50.39, *p* < 0.001, CFI = 0.98, TLI = 0.90, RMSEA = 0.052 (90% confidence interval = 0.039–0.067). The constrained model with all stability and predictive paths set equal for boys and girls was significantly worse than the unconstrained model, χ^2^(18) = 49.03, *p* < 0.001, CFI = 0.98, TLI = 0.92, RMSEA = 0.032 (90% confidence interval = 0.021–0.043), indicating gender differences. The stability of overt aggression from grade 8 to 9 and the paths from grade 8 popularity to popularity goal and overt aggression in grade 9 differed significantly by gender.

Next, a series of models was compared with each combination of these pathways constrained or freely estimated. There was no significant difference in model fit between the unconstrained model and the model with all paths constrained except for the stability of overt aggression from grade 8 to 9 and the predictive path of grade 8 popularity to grade 9 overt aggression, χ^2^(34) = 73.94, *p* < 0.001, CFI = 0.98, TLI = 0.94, RMSEA = 0.026 (90% confidence interval = 0.018–0.035). All stability paths for popularity and popularity goal were significant, *ps* < 0.001. Popularity in grades 7 and 8 was positively associated with popularity goal 1 year later (*βs* > 0.08, *ps* < 0.03). Popularity in grade 7 positively predicted overt aggression in grade 8 (*βs* > 0.05, *ps* = 0.042). For boys (but not for girls), grade 8 popularity was positively associated with grade 9 overt aggression (*β* = 0.27, *p* < 0.001). Overt aggression was stable from grade 8 to 9 for boys (*β* = 0.42, *p* < 0.001), but not for girls. Without considering gender differences, overt aggression in grade 7 was positively associated with popularity in grade 8 (*β* = 0.07, *p* < 0.01); however, this path was no longer significant in the model once accounting for gender differences.

#### Relational aggression

The model resulted in a similar pattern as the model for overt aggression (Fig. 1). The fit of the model was adequate, χ^2^(9) = 31.09, *p* < 0.001, CFI = 0.99, TLI = 0.95, RMSEA = 0.038 (90% confidence interval = 0.024–0.053). The fit of the unconstrained model, χ^2^(18) = 38.26, *p* = 0.004, CFI = 0.99, TLI = 0.96, RMSEA = 0.026 (90% confidence interval = 0.014–0.037), was significantly better than the model with all paths constrained, χ^2^(36) = 101.15, *p* < 0.001, CFI = 0.97, TLI = 0.93, RMSEA = 0.033 (90% confidence interval = 0.025–0.040), indicating gender differences. The model with all paths constrained except for the predictive path from grade 7 popularity to grade 8 relational aggression yielded the best fit, χ^2^(35) = 58.78, *p* = 0.007, CFI = 0.99, TLI = 0.97, RMSEA = 0.020 (90% confidence interval = 0.010–0.029). All stability paths were significant, *ps* < 0.001. Although popularity in grade 7 was positively associated with grade 8 relational aggression for both boys (*β* = 0.14, *p* = 0.01) and girls (*β* = 0.41, *p* < 0.001), the association was significantly stronger for girls. Popularity in grade 8 also positively predicted grade 9 relational aggression for both genders (*βs* > 0.25, *p* < 0.001). For boys and girls, grade 7 popularity was positively associated with grade 8 popularity goal (*βs* > 0.14, *ps* < 0.001). Relational aggression in grade 7 was negatively associated with popularity goal in grade 8 for boys and girls (*βs* > −0.09, *ps* = 0.029).

#### Alcohol use

The fit of the model (see Fig. 2) was good, χ^2^(9) = 25.06, *p* = 0.003, CFI = 0.99, TLI = 0.95, RMSEA = 0.033 (90% confidence interval = 0.018–0.048). The test of gender differences showed that the fit of the fully constrained model, χ^2^(36) = 57.23, *p* = 0.014, CFI = 0.99, TLI = 0.97, RMSEA = 0.019 (90% confidence interval = 0.009–0.028), was not significantly worse than the unconstrained model, χ^2^(18) = 38.99, *p* = 0.003, CFI = 0.99, TLI = 0.94, RMSEA = 0.026 (90% confidence interval = 0.015–0.038), indicating no significant gender differences. All stability paths were significant, *ps* < 0.001. Popularity in grades 7 and 8 was positively associated with popularity goal (*βs* > 0.08, *ps* < 0.04) and alcohol use (*βs* > 0.10, *ps* < 0.01) 1 year later. Alcohol use in grade 7 was positively associated with grade 8 popularity (*β* = 0.06, *p* = 0.024). Popularity goal in grade 8 positively predicted grade 9 alcohol use (*β* = 0.13, *p* = 0.002).

#### Prosocial behavior

The model (Fig. 3) had adequate fit, χ^2^(9) = 37.75, *p* < 0.001, CFI = 0.99, TLI = 0.93, RMSEA = 0.044 (90% confidence interval = 0.030–0.058). No paths were moderated by gender, as the fit of the fully constrained model, χ^2^(36) = 73.99, *p* < 0.001, CFI = 0.98, TLI = 0.95, RMSEA = 0.025 (90% confidence interval = 0.021–0.043), was not worse than the fit of the fully unconstrained model, χ^2^(18) = 48.19, *p* < 0.001, CFI = 0.98, TLI = 0.92, RMSEA = 0.032 (90% confidence interval = 0.021–0.043). In this model, all stability paths were significant, *ps* < 0.001. Popularity in grade was positively associated with grade 8 popularity goal (*β* = 0.12, *p* < 0.001) and negatively associated with grade 8 prosocial behavior (*β* = −0.10, *p* = 0.002).

## Discussion

Popularity and popularity goal are deeply intertwined with antisocial behaviors in adolescence (e.g., Dawes and Xie 2014). In response, researchers have begun to call for interventions that highlight ways in which popular adolescents can achieve their status goals using prosocial behavior, rather than antisocial behavior (e.g., Ellis et al. 2016). To do this effectively, it is vital to understand how popularity and popularity goal may relate differently to both negative and positive behaviors over time. However, only a few studies have examined the links between popularity and popularity goal in a prospective longitudinal design. The existing longitudinal investigations have typically examined changes over a short time span (6 months or 1 year; Dawes and Xie 2017), which limits inferences of how popularity, popularity goal, and behaviors reinforce each other across adolescence. Furthermore, past studies have typically focused solely on links between popularity, goals, and aggression or alcohol use, and have not also included prosocial behaviors. The goal of this study was therefore to clarify how longitudinal links between popularity, popularity goal, and aggression, alcohol use and prosocial behaviors develop over a longer time span.

### Popularity and Popularity Goal

Separate models were conducted for two forms of aggression, alcohol use, and prosocial behavior. In each model, the bidirectional associations between popularity and popularity goal were examined. As expected, popularity in grade 7 positively predicted popularity goal in grade 8 in each model. In other words, youth who were popular in Grade 7 had a stronger popularity goal in grade 8. A similar pattern was found from grade 8 to 9, although not as robust (not significant in all four models). Consistent with Dawes and Xie (2014), popularity goal did not significantly predict subsequent popularity. Together, these results suggest that throughout (early) adolescence, popularity precedes popularity goal, rather than the reverse. Thus, popularity goal may not be sufficient to gain status, yet having status predicts more strongly striving to gain or maintain one’s status. Still, there is some evidence that popularity goal may eventually lead to elevated status, insofar as the goal leads to specific changes in behavior (Li and Hu 2018). In a sample of Chinese early adolescents, Li and Hu (2018) found that popularity goal longitudinally predicted popularity, mediated by social cognitions of prosocial behavior as an effective method of achieving status and actual prosocial behavior. Therefore, under certain circumstances, youth with elevated popularity goal may be able to achieve their goal.

Of note, the current study sheds light on these processes in a Western, but non-American, sample, which is important as the associations of popularity that have been found predominantly in American samples may not be the same in other areas of world (e.g., European or Asian samples; van den Berg et al. in press). For example, in a recent meta-analysis, van den Berg et al. (in press) found that the correlation between popularity and preference was weaker in European samples than in North American and Chinese samples. Future studies should examine for whom and under which conditions popularity goal may result in increased status, while considering potential cultural differences.

### Popularity, Aggression and Alcohol use

Past research has shown strong support for popularity predicting subsequent aggression (e.g., Ojanen and Findley-Van Nostrand 2014). To a lesser extent, aggression has been found to predict popularity (Cillessen and Mayeux 2004). The results support that popularity is indeed generally a stronger predictor of (overt and relational) aggression than the reverse. Of note, the path from popularity to aggression was stronger at both time points for relational aggression than overt aggression, which is consistent with past findings indicating an especially strong longitudinal relation between popularity and relational aggression (e.g., Cillessen and Mayeux 2004). The current study did find some support that overt aggression in grade 7 was associated with elevated popularity in grade 8. However, this path was no longer significant in the final model accounting for gender differences. Moreover, contrary to expectations, relational aggression was not positively associated with elevated popularity over time. Adolescents who are already popular may be more inclined than those who are not popular to use aggression, perhaps to maintain or defend their social status. On the other hand, adolescents who are not popular may be less willing to behave aggressively, as they may not have the social resources or stature to be aggressive without consequences or retaliation (e.g., van den Berg et al. 2019). Furthermore, popularity is related to a variety of other peer-valued characteristics (e.g., attractiveness, style, humor: Vaillancourt and Hymel 2006) and it appears that aggression alone may not be enough to predict youth’s elevated popularity.

Popularity is also associated with alcohol use in adolescence (e.g., Gommans et al. 2016). As expected, there was a bidirectional, positive association between popularity and alcohol use from grade 7 to 8. Popularity in grade 8 also predicted subsequent alcohol use in grade 9 (but grade 8 alcohol use did not predict grade 9 popularity). This study found support of reciprocal associations between popularity and alcohol use in early adolescence. On the one hand, adolescents who are more popular may be more likely to drink alcohol because they have access to it (e.g., invitations to parties) or to show maturity (Moffitt 1993). On the other hand, adolescents who drink may experience increases in popularity due to appearing more mature. In the current study, alcohol use was a predictor of popularity only from grade 7 to 8, indicating that youth’s alcohol use may be more effective for elevated popularity when they enter a new school (begin secondary school). As researchers have posited, entering a new school provides a unique chance for adolescents to establish their social standing (e.g., Dawes and Xie 2017). It is possible that once a hierarchy is formed, popularity remains a strong predictor of behavior, whereas behavior is no longer a consistent predictor of popularity.

Taken together, the results indicated more consistent support for popularity as a predictor of aggression and alcohol use, rather than behavior as a predictor of popularity. Popularity was positively associated with both forms of aggression and alcohol use over time. Although popularity was a stronger predictor of behavior than behavior was of popularity, behavior did predict subsequent popularity in some cases. Overt aggression in grade 7 was positively associated with popularity in grade 8; however, this effect was no longer significant once considering gender differences. There was some support of bidirectional associations between popularity and alcohol use, as alcohol use was positively associated with subsequent popularity. Alcohol use may be more likely to predict elevated popularity than aggression, as aggression is associated with both high and low levels of popularity (e.g., Stoltz et al. 2016). That is, aggression by itself may not be enough to predict elevated popularity, whereas alcohol use may be more novel to peers and a salient signal of maturity. Of note, this finding builds on the work by Dumas et al. (2017) that found that drinking alcohol predicted self-perceived popularity, by showing bidirectional associations between alcohol and peer-reported popularity. These findings suggest that drinking alcohol can both make youth feel popular and also be seen by their peers as popular, and indicate a potential concerning cyclical association between drinking alcohol and popularity.

Although the current study focuses on peer-reported popularity, youth’s own perceptions of their status are also important to consider. For example, Mayeux and Cillessen (2008) found that youth who were high in popularity, and also high in self-perceived popularity, were most likely to increase in aggression. Even though the current study suggests that aggression alone is typically not enough to predict elevated popularity, future research should consider whether aggression is associated with increases in self-perceived popularity. If youth perceive themselves as more popular after engaging in aggression (regardless of whether their peers agree that they are more popular), then it may be even more difficult to convince youth to reduce their aggression.

### Popularity Goal, Aggression, and Alcohol Use

Popularity goal was expected to predict subsequent aggression and alcohol use at both times, but support was only found for this hypothesis for alcohol use in grade 9. Contrary to expectations, popularity goal did not predict subsequent aggression. This is somewhat at odds with past research indicating links between popularity goal and aggression (e.g., Dumas et al. 2017), but consistent with research suggesting that popularity goal alone does not prospectively predict aggression when accounting for other variables (e.g., popularity; Dawes and Xie 2014). Acting aggressively to peers is not without risk, as youth may face retaliation or anger from their classmates. Perhaps some youth with high popularity goal attempt to use aggressive behaviors to increase their status, but quickly disengage from it if it does not successfully lead to increases in popularity.

A slightly different pattern emerged for drinking alcohol, as popularity goal in grade 8 predicted increases in alcohol use in grade 9. For both genders, drinking was lower in grade 8 (*Ms* < 0.13) than grade 9 (*M*s > 0.28). It is possible that drinking was not prevalent enough in grade 8, when youth are between the ages of 13 to 15, to be predicted by popularity goal in grade 7. Moreover, when drinking is still relatively uncommon, perhaps only youth with high levels of popularity have opportunities to drink, rather than those with a high popularity goal. However, as youth get older and drinking alcohol becomes more common, those who want to be popular (along with those who already are popular) appear particularly likely to drink alcohol. Despite the relatively low prevalence of alcohol use in this age group, it is particularly important to understand mechanisms that underlie alcohol use in early adolescence, as youth who begin to drink alcohol between ages 11–14 years are more likely to develop alcohol abuse or dependence (Zeigler et al. 2005). Moreover, research suggests that prevention programs on delaying substance use should target early adolescence (Gallimberti et al. 2015). This study suggests that prevention programs may want to specifically target early adolescents who are, or want to be, popular.

One reason why popularity goal may have predicted alcohol use, but not aggression, is because alcohol use was less common in this sample. In other words, during a time period where alcohol use is still relatively uncommon, the youth who choose to drink alcohol may be those who are particularly motivated to be popular. In early adolescence, aggression is a more prevalent behavior than alcohol use and may be driven by more factors than just popularity goal.

Although this association was not hypothesized, relational aggression in grade 7 was negatively associated with popularity goal in grade 8. This finding is inconsistent with prior speculation (e.g., Dumas et al. 2017) that relational aggression reaffirms social goals and leads to increases in popularity goal over time. Future research should further investigate the circumstances in which popularity goal and aggression are longitudinally linked, as the pattern of these associations is still unclear.

### Gender Differences

There were several mean level gender differences in terms of aggression and alcohol use. Boys scored higher on overt aggression in all grades, and their overt aggression was more stable than that of girls, whereas girls scored higher on relational aggression than boys in grades 8 and 9. In grade 7 only, boys were more likely than girls to drink alcohol. Yet, there were only a few gender differences regarding the associations of aggression and alcohol use with popularity and popularity goal. Popularity in grade 8 predicted elevated overt aggression in grade 9 for boys only, whereas popularity in grade 7 predicted elevated overt aggression in grade 8 for both boys and girls. Furthermore, popularity predicted high levels of relational aggression for both boys and girls. These findings are consistent with past research suggesting that, while overt or physical forms of aggression may be somewhat more common among boys than girls, relational aggression is used by both girls and boys (e.g., Card et al. 2008; Rose et al. 2011). Whereas popularity predicted elevated levels of relational aggression for both genders, it must be noted that the effect of popularity on relational aggression was much stronger for girls than boys, and was the strongest cross-path (*β* = 0.41) in all models tested. Taken together, this suggests that overt aggression was more characteristic of boys, whereas the association between popularity and relational aggression was stronger for girls than boys. Nonetheless, the findings only highlighted a few gender differences, suggesting that the prospective associations between popularity, popularity goal, and aggression and alcohol use are largely the same for boys and girls.

### Popularity, Popularity Goal, and Prosocial Behavior

As a subset of youth is popular and prosocial (e.g., de Bruyn and Cillessen 2006; Rodkin et al. 2000), the current study also tested the longitudinal links between popularity, popularity goal, and prosocial behavior. The findings for concurrent associations between prosociality and popularity are mixed, while prosociality and popularity motivations appear to be negatively associated (e.g., Cillessen et al. 2014). The current study showed that popularity goal was unrelated to prosocial behavior, whereas popularity was negatively associated with prosocial behavior over time. Of note, this finding was only present from grade 7 to 8, and may reflect the social reshuffling that occurs after adolescents enter a new school (e.g., Dawes and Xie 2017). In other words, high popularity during the 1st year of secondary school (grade 7) may lead to decreases in prosocial or cooperative behavior (and increases in aggression or substance use) as adolescents attempt to establish social dominance.

Resource control theory, however, suggests that a subset of youth (i.e., bistrategics) implement both prosocial and aggressive behaviors to gain resources (e.g., popularity; Hawley 2003; Wurster and Xie 2014). Although bistrategics may successfully use both aggression and prosocial behavior to maintain status (e.g., Hawley 2003), this study suggests that aggression and alcohol use are more strongly linked to popularity and popularity goal over time than prosocial behaviors. One explanation could be that popular adolescents may sometimes display prosocial behavior, but may not have a reputation amongst peers as someone who behaves prosocially or helps classmates, given their concurrent antisocial behaviors. Alternatively, prosocial behavior measured with “helping others” may not be close enough conceptually to prosocial resource control. Whereas prosocial resource control shows overlap with “plain” prosocial behavior (e.g., helping, cooperation), prosocial resource control is aimed at gaining access to resources (e.g. explaining why their idea is good: Hawley 2003), and thus may be related differently to popularity variables than “plain” prosocial behavior. Moreover, there is burgeoning evidence that distinct forms of prosocial behavior serve different functions (e.g., proactive, reactive, altruistic), and that these forms are differentially related to popularity (Findley-Van Nostrand and Ojanen 2018). Furthermore, previous research indicates that cooperative behaviors may only be successful at gaining resources in certain circumstances (e.g., when everyone has access to resources), but not when individuals are competing for resources (Pellegrini 2008).

### Limitations and Future Directions

The current study contributes to the growing literature on the associations between popularity, popularity goal, and social behavior in adolescence by investigating these associations in a multi-informant, 3-year prospective longitudinal study. Nonetheless, this study had some limitations. Popularity goal and alcohol use were measured with single-item self-reports. The measures used are consistent with other recent studies on popularity goal (e.g., Dawes and Xie 2014; Wright et al. 2014) and health-risk behaviors (e.g., alcohol use; Osgood et al. 2013). Nevertheless, future research should test whether multi-item measures of popularity goals and alcohol use yield a similar pattern of longitudinal effects. In addition, there are other forms of aggression (e.g., proactive aggression, reactive aggression) and substance use (e.g., smoking) that should be considered in future research.

Furthermore, these findings relate to one type of social goal. However, youth have other drives (e.g., communal goals) that may be differentially related to behaviors and popularity. For example, adolescents with high status goals who also have high communal goals behave differently than adolescents who only care about being popular (e.g., Ojanen and Findley-Van Nostrand 2014). More longitudinal research is needed to elucidate how other social goals relate to status and behaviors over time. Moreover, potential moderators other than gender were not examined. The main goal of this study was to examine the temporal direction of the associations among popularity, popularity goal, and behaviors longitudinally, as most research has examined these associations concurrently or short-term. However, previous research suggests that there may be other moderators at play. For example, Dawes and Xie (2014) found that the interaction of popularity, popularity goal, and social aggression in the fall of grade 6 predicted popularity in the next semester. Although this study serves as a foundation for the longitudinal pathways of adolescents’ popularity, popularity goal, and behaviors, future research should build on this with longitudinal designs that consider how popularity and popularity goal may interact to predict behaviors.

The associations between popularity, popularity goal, and behavior may also be related to the (mis)match between youth’s popularity and popularity goal. In other words, youth who want to be popular (and also are popular) may have different behavioral profiles than youth who want to be popular but are not popular (i.e., “wannabes”: Breslend et al. 2018). Future research could identify subtypes of adolescents based on their overlap of popularity and popularity goal and compare their adjustment (e.g., aggression) over time.

## Conclusion

Past research has demonstrated the important roles that popularity and popularity goal play in youth’s behavior. However, most of the extant literature has focused primarily on the associations between popularity, popularity goal, and aggression or alcohol use. Moreover, there is a dearth of longitudinal studies with more than two time points, which limits understanding of how these associations develop across adolescence. The current study built on past research by examining the developmental pathways and prospective associations among popularity, popularity goal, and adolescents’ aggression, alcohol use and prosocial behaviors over 3 years. In general, the results demonstrate that popularity is a consistent predictor of elevated popularity goal, as well as higher engagement in risk behaviors (i.e., alcohol use) and aggression. This study adds to previous research indicating that there are risks associated with popularity (e.g., Mayeux et al. 2008; Schwartz and Gorman 2011). In order for research to address this association, it is important to examine how these processes unfold over time, especially given the influence that popular adolescents have over their peers (e.g., Teunissen et al. 2012). By understanding the temporal direction of popularity, popularity goal, and social behaviors, researchers can better inform prevention and intervention efforts. Furthermore, recent interventions efforts have focused on incorporating the unique role of status and status goals in adolescence, and explored ways to satisfy these needs through engagement in prosocial behaviors (e.g., Ellis et al. 2016). This study underscores the importance of developing interventions that carefully consider the dynamics of popularity, goals, and behaviors, as popularity predicted elevated levels of aggression and alcohol use but lower levels of prosocial behavior.

## Appendix

Tables 3–6

**Table 3:** Model fit statistics and chi-square difference test comparing fit of unconstrained and constrained models for overt aggression

Model		χ^2^ (*df*)	Δχ^2^ (*df*)	*p*	CFI	TLI	RMSEA
	Step 1						
1	Fully unconstrained	49.03 (18)	–	–	0.98	0.92	0.032
2	All path coefficients constrained	124.42 (36)	75.39 (18)	<0.001	0.95	0.88	0.038
	Step 2						
2a	Constrain G7 Pop → G8 Pop	49.05 (19)	0.02 (1)	0.893	0.98	0.92	0.031
2b	Constrain G8 Pop → G9 Pop	49.03 (19)	0.00 (1)	0.994	0.98	0.92	0.031
2c	Constrain G7 Goal → G8 Goal	49.15 (19)	0.12 (1)	0.739	0.98	0.92	0.031
2d	Constrain G8 Goal → G9 Goal	50.55 (19)	1.52 (1)	0.218	0.98	0.92	0.031
2e	Constrain G7 Overt → G8 Overt	50.74 (19)	1.71 (1)	0.191	0.98	0.92	0.032
2f	Constrain G8 Overt → G9 Overt	69.84 (19)	20.81 (1)	<0.001	0.97	0.87	0.040
2g	Constrain G7 Pop → G8 Goal	51.33 (19)	2.30 (1)	0.130	0.98	0.92	0.032
2h	Constrain G7 Pop → G8 Overt	51.26 (19)	2.23 (1)	0.136	0.98	0.92	0.032
2i	Constrain G7 Goal → G8 Pop	50.57 (19)	1.54 (1)	0.215	0.98	0.92	0.031
2j	Constrain G7 Goal → G8 Overt	49.42 (19)	0.39 (1)	0.532	0.98	0.92	0.031
2k	Constrain G7 Overt → G8 Pop	51.02 (19)	1.99 (1)	0.159	0.98	0.92	0.032
2l	Constrain G7 Overt → G8 Goal	49.28 (19)	0.25 (1)	0.621	0.98	0.92	0.031
2m	Constrain G8 Pop → G9 Goal	52.91 (19)	3.88 (1)	0.049	0.98	0.91	0.033
2n	Constrain G8 Pop → G9 Overt	59.85 (19)	10.82 (1)	0.001	0.98	0.90	0.036
2o	Constrain G8 Goal → G9 Pop	50.38 (19)	1.35 (1)	0.245	0.98	0.92	0.031
2p	Constrain G8 Goal → G9 Overt	52.26 (19)	3.23 (1)	0.072	0.98	0.92	0.032
2q	Constrain G8 Overt → G9 Pop	49.05 (19)	0.02 (1)	0.907	0.98	0.92	0.031
2r	Constrain G8 Overt → G9 Goal	50.58 (19)	1.55 (1)	0.213	0.98	0.92	0.031
	Step 3				0.98		
3	New baseline: constrain all paths but G8 Overt → G9 Overt, G8 Pop → G9 Goal, G8 Pop → G9 Overt	71.65 (33)	–	–	0.98	0.94	0.026
	Step 4						
3a	Constrain all paths but G8 Overt → G9 Overt	92.99 (35)	21.34 (2)	<0.001	0.97	0.92	0.031
3b	Constrain all paths but G8 Pop → G9 Goal	121.94 (35)	50.29 (2)	<0.001	0.95	0.88	0.038
3c	Constrain all paths but G8 Pop → G9 Overt	93.64 (35)	21.99 (2)	<0.001	0.97	0.92	0.032
3d	Constrain all paths but G8 Overt → G9 Overt, G8 Pop → G9 Goal	90.65 (34)	19.00 (1)	<0.001	0.97	0.92	0.031
3e	Constrain all paths but G8 Pop → G9 Goal, G8 Pop → G9 Overt	91.23 (34)	19.58 (1)	<0.001	0.97	0.92	0.032
3f	Final model	73.94 (34)	2.29 (1)	0.130	0.98	0.94	0.026

**Table 4:** Model fit statistics and chi-square difference test comparing fit of unconstrained and constrained models for relational aggression

Model		χ^2^ (*df*)	Δχ^2^ (*df*)	*p*	CFI	TLI	RMSEA
	Step 1						
1	Fully unconstrained	38.26 (18)	–	–	0.99	0.96	0.026
2	All path coefficients constrained	101.15 (36)	62.89 (18)	<0.001	0.97	0.93	0.033
	Step 2						
2a	Constrain G7 Pop → G8 Pop	38.87 (19)	0.61 (1)	0.434	0.99	0.96	0.025
2b	Constrain G8 Pop → G9 Pop	38.69 (19)	0.43 (1)	0.515	0.99	0.96	0.025
2c	Constrain G7 Goal → G8 Goal	38.39 (19)	0.13 (1)	0.720	0.99	0.96	0.025
2d	Constrain G8 Goal → G9 Goal	39.64 (19)	1.38 (1)	0.240	0.99	0.96	0.025
2e	Constrain G7 Rel → G8 Rel	39.63 (19)	1.37 (1)	0.243	0.99	0.96	0.025
2f	Constrain G8 Rel → G9 Rel	41.04 (19)	2.78 (1)	0.095	0.99	0.86	0.026
2g	Constrain G7 Pop → G8 Goal	40.45 (19)	2.19 (1)	0.139	0.99	0.96	0.026
2h	Constrain G7 Pop → G8 Rel	59.19 (19)	20.93 (1)	<0.001	0.98	0.92	0.035
2i	Constrain G7 Goal → G8 Pop	40.39 (19)	2.13 (1)	0.144	0.99	0.96	0.026
2j	Constrain G7 Goal → G8 Rel	39.26 (19)	1.00 (1)	0.319	0.99	0.96	0.025
2k	Constrain G7 Rel → G8 Pop	41.94 (19)	3.68 (1)	0.055	0.99	0.95	0.027
2l	Constrain G7 Rel → G8 Goal	38.98 (19)	0.72 (1)	0.396	0.99	0.96	0.025
2m	Constrain G8 Pop → G9 Goal	41.01 (19)	2.75 (1)	0.097	0.99	0.96	0.026
2n	Constrain G8 Pop → G9 Rel	38.85 (19)	0.59 (1)	0.442	0.99	0.96	0.025
2o	Constrain G8 Goal → G9 Pop	39.48 (19)	1.22 (1)	0.270	0.99	0.96	0.025
2p	Constrain G8 Goal → G9 Rel	38.66 (19)	0.40 (1)	0.526	0.99	0.96	0.025
2q	Constrain G8 Rel → G9 Pop	38.91 (19)	0.65 (1)	0.421	0.99	0.96	0.025
2r	Constrain G8 Rel → G9 Goal	38.26 (19)	0.00 (1)	0.993	0.99	0.96	0.025
	Step 3						
3	Final model	58.78 (35)	20.52 (17)	0.249	0.99	0.97	0.020

**Table 5:** Model fit statistics and chi-square difference test comparing fit of unconstrained and constrained models for alcohol use

Model	Step 1	χ^2^ (*df*)	Δχ^2^ (*df*)	*p*	CFI	TLI	RMSEA
1	Fully unconstrained	38.99 (18)	–	–	0.99	0.94	0.026
2	All path coefficients constrained	57.23 (36)	18.24 (18)	0.440	0.99	0.97	0.019

**Table 6:** Model fit statistics and chi-square difference test comparing fit of unconstrained and constrained models for prosocial behavior

Model	Step 1	χ^2^ (*df*)	Δχ^2^ (*df*)	*p*	CFI	TLI	RMSEA
1	Fully unconstrained	48.19 (18)	–	–	0.98	0.92	0.032
2	All path coefficients constrained	73.99 (36)	25.80 (18)	0.104	0.98	0.95	0.025
